# Supplementary material for: External Validation of the Dutch SOURCE Survival Prediction Model in Belgian Metastatic Oesophageal and Gastric Cancer Patients
Source: Cancers (Basel). 2020 Mar 31;12(4):834. doi: 10.3390/cancers12040834 (PMC7225946; doi:10.3390/cancers12040834)
Supplement: Supplementary file 1 [file cancers-12-00834-s001.pdf]

Supplementary Materials: External Validation of the Dutch SOURCE Survival Prediction Model in Belgian Metastatic Oesophageal and Gastric Cancer Patients

J.J. van Kleef, H.G. van den Boorn, R.H.A. Verhoeven, K. Vanschoenbeek, A. Abu-Hanna, A.H. Zwinderman, M.A.G. Sprangers, M.G.H. van Oijen, H. De Schutter and H.W.M. van Laarhoven

1. Supplementary Methods

1.1. The Belgian Cancer Registry

Data obtained from the BCR included age at diagnosis, sex, PS, year of diagnosis, topography, morphology, differentiation grade, (c/p)TNM stage, location of metastases, and information on systemic oncological, radiotherapeutic and surgical procedures up to six months after diagnosis.

Patient and tumour characteristics were collected from the standard cancer registration database, which relies on notifications from both the clinical (oncology care programs) and pathological (laboratories for pathological anatomy) network.

Data regarding treatment were derived from reimbursement claims of health insurance companies as gathered by the Intermutualistic Agency. These data were linked to the BCR cancer registration data using the National Social Security Number (NSSN) as unique patient identifier, according to existing authorizations. All reimbursed diagnostic, therapeutic and pharmaceutical procedures from the in- and outpatient setting were available for a period from one year before until five years after diagnosis for each patient, limited to the end of 2017. Similarly, NSSN was used to retrieve information about vital status' from the Crossroads Bank of Social Security, updated until 1 July 2017.

Data regarding the number of metastatic sites and their localisation was retrieved from the diagnostic codes (ICD-9-CM: 196, 197 and 198) within the time frame six months before until six months after incidence date as present in hospital discharge data available at BCR for this patient selection. These data were available for 57.9% of the patients. The use of BCR data for scientific purposes is regulated by Belgian law, excluding the need for written informed consent for this study.[17]

1.2. Patient selection

Patients who did not have adeno- or squamous cell carcinomas (SCC), who had a cT0/Tis or tumour location C15.1 were excluded given SOURCE selection criteria. Patients with metastases only in lymph nodes of the head and neck area were also excluded. Additionally, patients who died within 14 days after diagnosis were excluded, given that prediction models are then not likely applied.

2. Supplementary Results

Model re-estimation

Parameters of the re-estimated oesophageal and gastric cancer model using BCR data, are given in Supplementary Table 1. Internal validation of the oesophageal cancer model at six months follow-up showed a c-index of 0.68 (0.66–0.69), an intercept of 0.00 (0.00–0.01), a slope of 0.97 (0.97–0.98) and an absolute mean error of 2.6% (Supplementary Figure 1). Mean differences between predicted and observed OS were +1.1%, see Supplementary Table 2. Internal validation of the gastric cancer model at six months follow-up showed a c-index of 0.68 (0.66–0.69), an intercept of -0.04 (-0.05–-0.04), a slope of 1.05 (1.05–1.06) and an absolute mean error of 2.3%, see Supplementary Figure 1. Mean differences between predicted and observed OS were also +2.3% (Supplementary Table 2).

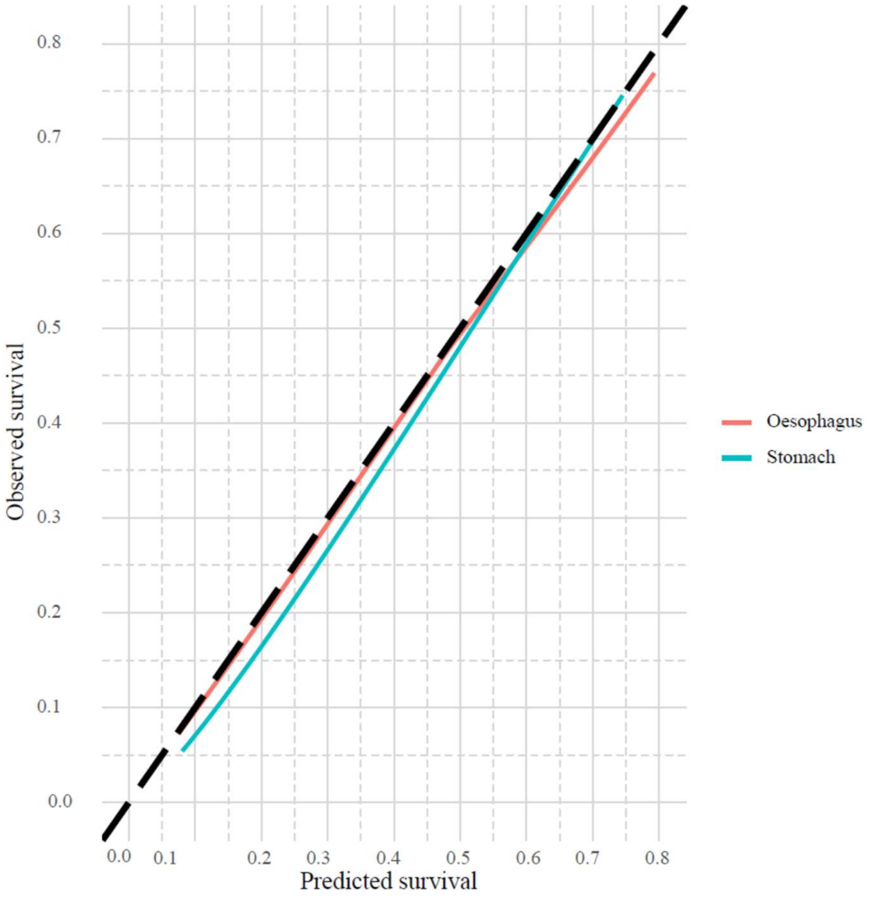

Figure S1. Calibration plot of predicted versus observed six-month overall survival for patients with oesophageal cancer (red line) and gastric cancer (blue line). Results are shown from the re-estimated Belgian model.

Table S1. Re-estimation of the SOURCE prediction model for overall survival in Belgium patients with metastatic oesophageal and gastric cancer.

Metastatic Oesophageal Cancer Model		Metastatic Gastric Cancer Model	
**Covariate**	**HR 95% CI**	**Covariate**	**HR 95% CI**
Age at diagnosis	1.01 (1.00–1.03)	Age at diagnosis	1.01 (1.01–1.02)
cT category		Gender	
T1	1	Male	1
T2	1.12 (0.91–1.59)	Female	0.92 (0.82–1.02)
T3	1.17 (0.91–1.51)	cT category	
T4	1.43 (1.09–1.89)	T1	1
TX	1.52 (1.17–1.96)	T2	1.27 (0.90–1.78)
cN category		T3	1.37 (1.01–1.86)
N0	1	T4	1.29 (0.95–1.76)
N1	0.97 (0.86–1.09)	TX	1.53 (1.14–2.04)
N2	1.01 (0.89–1.14)	cN category	
N3	1.26 (1.09–1.47)	N0	1
Tumour differentiation grade		N1	1.08 (0.93–1.25)
G1	1	N2	1.12 (0.97–1.30)
G2	1.21 (1.04–1.42)	N3	1.28 (1.01–1.61)
G3	1.20 (1.02–1.4)	Tumour differentiation grade	
G4	1.03 (0.80–1.33)	G1	1
Number of metastatic sites	0.97 (0.90–1.04)	G2	1.12 (0.92–1.37)
Only distant lymph node metastasis		G3	1.29 (1.07–1.54)
No	1	G4	1.25 (0.87–1.81)
Yes	0.79 (0.68–0.92)	Number of metastatic sites	1.18 (1.10–1.26)
Morfology		Only distant lymph node metastasis	
Adenocarcinoma	1	No	1
Squamous cell	1.02 (0.91–1.14)	Yes	1.24 (1.01–1.53)
Other	1.91 (1.32–2.76)	Intra-thoracic lymph node metastasis	
Tumour location		No	1
Cervical	1	Yes	0.88 (0.75–1.03)
Upper thoracic	1.04 (0.57–1.93)	Intra-abdominal lymph node metastasis	
Mid-thoracic	1.09 (0.60–1.96)	No	1
Lower thoracic	1.18 (0.66–2.12)	Yes	0.73 (0.64–0.83)
Overlapping lesion	1.35 (0.49–3.72)	Initial treatment	
Esophagus NOS	1.13 (0.63–2.02)	None	1

Junction	0.98 (0.54–1.77)
Lymph node metastasis in head/neck area	
No	1
Yes	0.68 (0.60–0.77)
Intra-thoracic lymph node metastasis	
No	1
Yes	0.77 (0.57–1.04)
Intra-abdominal lymph node metastasis	
No	1
Yes	0.76 (0.58–0.99)
Liver metastasis	
No	1
Yes	1.11 (1.00–1.23)
Peritoneal metastasis	
No	1
Yes	1.167 (1.04–1.31)
Initial treatment	
None	1
Chemotherapy	0.30 (0.10–0.85)
Radiotherapy (primary tumour)	0.81 (0.20–3.33)
Chemoradiation	0.04 (0.01–0.43)
Chemotherapy plus short term radiation	0.20 (0.06–0.74)
Stent	1.47 (0.37–5.89)
Other	0.23 (0.06–0.82)
Initial treatment chemotherapy	
* Age at diagnosis	0.99 (0.98–1.01)
* Number of metastatic sites	1.17 (1.09–1.25)
* Intra-thoracic lymph node metastasis	1.12 (0.81–1.54)
* Intra-abdominal lymph node metastasis	1.09 (0.81–1.47)
Radiotherapy (primary tumour)	
* Age at diagnosis	0.98 (0.97–1.00)
* Number of metastatic sites	1.13 (1.01–1.26)
* Intra-thoracic lymph node metastasis	1.19 (0.72–1.98)
* Intra-abdominal lymph node metastasis	1.42 (0.87–2.32)
Chemoradiation	
* Age at diagnosis	1.02 (0.98–1.05)
Chemotherapy	0.55 (0.23–1.28)
Radiotherapy (primary tumour)	5.97 (0.62–57.76)
Chemotherapy plus short term radiation	0.09 (0.01–1.86)
Resection (primary tumour)	0.31 (0.07–1.36)
Other	0.24 (0.04–1.55)
Initial treatment chemotherapy	
* Age at diagnosis	1.00 (0.99–1.01)
* Number of metastatic sites	0.96 (0.88–1.04)
Initial treatment radiotherapy (primary tumour)	
* Age at diagnosis	0.98 (0.94–1.01)
* Number of metastatic sites	0.97 (0.75–1.25)
Initial treatment resection (primary tumour)	
* Age at diagnosis	1.00 (0.98–1.02)
* Number of metastatic sites	1.04 (0.84–1.28)
Initial treatment chemotherapy plus short term radiation	
* Age at diagnosis	1.02 (0.98–1.06)
* Number of metastatic sites	1.12 (0.83–1.5)
Initial treatment 'other'	
* Age at diagnosis	1.02 (0.99–1.04)
* Number of metastatic sites	0.88 (0.73–1.07)

* Number of metastatic sites	1.37 (1.03–1.82)
* Intra-thoracic lymph node metastasis	1.20 (0.52–2.76)
* Intra-abdominal lymph node metastasis	1.53 (0.66–3.53)
Chemotherapy plus short term radiation	
* Age at diagnosis	1.00 (0.98–1.02)
* Number of metastatic sites	1.10 (1.01–1.19)
* Intra-thoracic lymph node metastasis	1.24 (0.81–1.91)
* Intra-abdominal lymph node metastasis	1.15 (0.80–1.68)
Stent	
* Age at diagnosis	0.99 (0.97–1.01)
* Number of metastatic sites	1.08 (0.99–1.19)
* Intra-thoracic lymph node metastasis	1.05 (0.68–1.61)
* Intra-abdominal lymph node metastasis	1.12 (0.76–1.65)
Other	
* Age at diagnosis	1.00 (0.99–1.02)
* Number of metastatic sites	1.14 (1.03–1.26)
* Intra-thoracic lymph node metastasis	1.18 (0.77–1.80)
* Intra-abdominal lymph node metastasis	0.95 (0.63–1.42)

Table S2. Calibration and discriminative ability of the re-estimated model for the Belgium population.

Endpoint	Intercept	Slope	Absolute Error (%)	Predicted-Observed Survival (%)	C-Index
			**Oesophageal cancer model**		
6-month survival	0.00 (-0.00–0.01)	0.97 (0.97–0.98)	2.6 (2.5–2.6)	1.1 (1.0–1.2)	0.68 (0.66–0.69)
			**Gastric cancer model**		
6-month survival	-0.04 (-0.05–-0.04)	1.05 (1.05–1.06)	2.3 (2.3–2.3)	2.3 (2.1–2.4)	0.68 (0.66–0.69)

© 2020 by the authors. Licensee MDPI, Basel, Switzerland. This article is an open access article distributed under the terms and conditions of the Creative Commons Attribution (CC BY) license (http://creativecommons.org/licenses/by/4.0/).
